# Cardiovascular Active Peptides of Marine Origin with ACE Inhibitory Activities: Potential Role as Anti-Hypertensive Drugs and in Prevention of SARS-CoV-2 Infection

**DOI:** 10.3390/ijms21218364

**Published:** 2020-11-07

**Authors:** Marco Festa, Clementina Sansone, Christophe Brunet, Fabio Crocetta, Luisa Di Paola, Michele Lombardo, Antonino Bruno, Douglas M. Noonan, Adriana Albini

**Affiliations:** 1 IRCCS MultiMedica, 30138 Milan, Italy; marcomariogiacomo.festa@multimedica.it (M.F.); michele.lombardo@multimedica.it (M.L); douglas.noonan@uninsubria.it (D.M.N.); 2 Department of Marine Biotechnology, Stazione Zoologica Anton Dohrn, 80121 Naples, Italy; clementina.sansone@szn.it (C.S.); christophe.brunet@szn.it (C.B.); 3 Department of Integrative Marine Ecology, Stazione Zoologica Anton Dohrn, 80121 Naples, Italy; fabio.crocetta@szn.it; 4 Unit of Chemical-physics Fundamentals in Chemical Engineering, Department of Engineering, Università Campus Bio-Medico di Roma, 00128 Rome, Italy; l.dipaola@unicampus.it; 5 Laboratory of Immunology and General Pathology, Department of Biotechnology and Life Sciences, University of Insubria, 21100 Varese, Italy

**Keywords:** ACE inhibitors, marine peptides, sea organisms, COVID-19, renin-angiotensin-aldosterone system, ACE2, cardiometabolic syndrome, hypertension

## Abstract

Growing interest in hypertension—one of the main factors characterizing the cardiometabolic syndrome (CMS)—and anti-hypertensive drugs raised from the emergence of a new coronavirus, SARS-CoV-2, responsible for the COVID19 pandemic. The virus SARS-CoV-2 employs the Angiotensin-converting enzyme 2 (ACE2), a component of the RAAS (Renin-Angiotensin-Aldosterone System) system, as a receptor for entry into the cells. Several classes of synthetic drugs are available for hypertension, rarely associated with severe or mild adverse effects. New natural compounds, such as peptides, might be useful to treat some hypertensive patients. The main feature of ACE inhibitory peptides is the location of the hydrophobic residue, usually Proline, at the C-terminus. Some already known bioactive peptides derived from marine resources have potential ACE inhibitory activity and can be considered therapeutic agents to treat hypertension. Peptides isolated from marine vertebrates, invertebrates, seaweeds, or sea microorganisms displayed important biological activities to treat hypertensive patients. Here, we reviewed the anti-hypertensive activities of bioactive molecules isolated/extracted from marine organisms and discussed the associated molecular mechanisms involved. We also examined ACE2 modulation in sight of SARS2-Cov infection prevention.

## 1. Introduction

Plant-derived molecules, such as aspirin from willow bark, Taxol from yew bark, digitoxin from digital seeds, and many others, have been employed for decades in medicine [1,2,3]. However, when compared to the terrestrial counterpart, the marine environment remains under-investigated as a source of drugs of pharmaceutical interests. This is a missed opportunity, considering that oceans cover 70% of the earth surface, hosting a wide range of different habitats/environments with high biodiversity, that in turn represent a valuable source of potentially beneficial agents for human health, including several bioactive compounds derived from primary or secondary metabolism pathways [4,5,6]. Secondary metabolites originated from marine organisms exhibit relevant activities, including defense against predators and protection against pathogens [7]. The unique biodiversity of marine organisms, characterized by an elevated chemodiversity, is attracting the interest of the scientific community towards the identification of novel agents for biomedical applications and biotechnology purposes. While “terrestrial” drugs are mostly of phytochemical origin, marine bioactive compounds include more than 20 molecules of animal origin, mainly isolated from invertebrates, such as ascidians, gastropods, polychaetas, and from vertebrates, such as fishes, that are registered or studied in phases II and III clinical trials for drug development [7]. Given the multiple functions of secondary metabolites, these agents can have great pharmacological potentials as oncological treatments or preventive. Further fields of application for these agents include infectious diseases (antibiotics) [8], analgesics [9], and they could find applications in the treatment of metabolic syndromes, cardiometabolic disorders (CMD), diabetes [10], and hypertension.

Hypertension is characterized by elevated blood pressure and functional and structural vascular and cardiac anomalies that further damage target organs (heart, kidneys, brain, vessels) and cause premature morbidity and mortality [11].

The renin–angiotensin–aldosterone system (RAAS) regulates the blood pressure [12,13]. Renin, produced by kidneys to counteract decrease blood flow, cleaves its substrate hormone angiotensinogen, generating the decapeptide Angiotensin I that is in turn cleaved by Angiotensin-converting enzyme (ACE) to produce the angiotensin II (Ang II) [12,13]. Ang II binds to its receptor on the cell surface AT1R inducing vasoconstriction, renal sodium (Na^+^) reabsorption, and Aldosterone secretion, thereby increasing blood pressure and bringing a higher flux to the kidneys [12,13]. The angiotensin-converting enzyme 2 (ACE2) cleaves Ang II to Ang (1–7), binding to MES, and causes vasodilation and cardioprotection. ACE2 is a “protective” enzyme to downregulate high blood pressure and inflammation [12,13].

Different classes of synthetic drugs are used to treat hypertension such as direct inhibitors of the renin-angiotensin system (ACE inhibitors, angiotensin receptor blockers), beta-adrenergic antagonists, diuretics, inhibitors of the calcium channel blockers and aldosterone receptors. Some of these drugs infrequently give rise to mild or severe side effects, limiting their practice in medicine. Major classes of anti-hypertensive drugs directed to the angiotensin system include ACE inhibitors (captopril, enalapril, lisinopril, perindopril, ramipril, and others) and angiotensin receptor blockers (ARBs) (losartan, olmesartan, valsartan, telmisartan).

Diverse marine animal organisms, such as fishes, shellfishes, cephalopods, seaweeds, and macro- and micro-algae, account as a relevant source of proteins and bioactive peptides, some of which are potentially endowed with anti-hypertensive, anticoagulant, antithrombotic, and hypocholesterolemic activities [4]. For example, fractions and subfractions of the enzyme hydrolysate of the jellyfish Rhopilema esculentum have been reported to hinder ACE activity at IC50 starting from 1.28 mg mL^−1^ 0.16 mg mL^−1^, respectively [14].

Anti-ACE peptides can be derived from different natural sources that include animals or algae and plants and present with interesting anti-hypertensive effects comparable to the synthetic molecules [15]. 

A recent coronavirus family member, namely SARS-CoV-2, responsible for the COVID19 pandemic [16], gains cell entry through the interactions between the Spike viral protein and the Angiotensin-converting enzyme 2 (ACE2) [17]. Anti-ACE2 marine peptides with antiviral activity could address this novel challenge.

Patients with comorbidities, in particular those affected by cardiovascular diseases [10], hypertension, and diabetes, have been reported to worsen their clinical conditions when infected by SARS-CoV-2 significantly. ACE inhibitors’ role can also be very relevant for the development or protection of the clinical pattern of Covid-19 syndrome [18,19].

Here, we review and discuss the ACE inhibitory activities of peptides derived from marine sources, and summarize the mechanisms of action of these peptides, to be potentially employed as therapeutic agents for hypertensive patients. Finally, we show preliminary observations on the docking of peptides of marine origin with the ACE2 molecule, both in apo form and in complex with SARS-CoV2 spike receptor binding domain (RBD).

## 2. Hypertension

**Figure 1:**
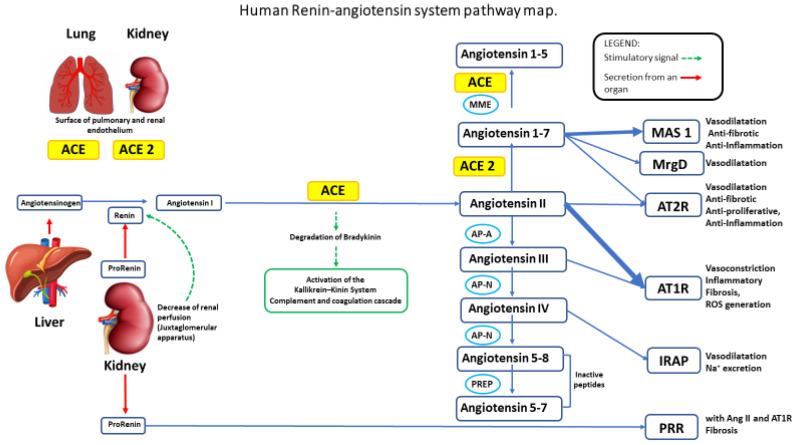
Cartoon summarizing the molecular factors involved in the Renin–angiotensin–aldosterone system (RAAS) as a system. The enzyme Renin cleaves its substrate angiotensinogen (AGT), generating the decapeptide Angiotensin I that is cleaved by Angiotensin-converting enzyme (ACE) to produce the angiotensin II (Ang II). Ang II activates its AT1 receptor (AT1R), the principal receptor that mediates most of the known actions of Ang II in the kidney, including vasoconstriction, renal sodium (Na^+^) reabsorption, and Aldosterone secretion, increasing blood pressure. In addition to (ACE)/Ang II/AT1R and AT2R axis, other signaling pathways in the RAAS, such as ACE2/angiotensin-(1–7), improve vasodilatation through MAS and Ang IV/IRAP, and other active peptides of the RAAS, with physiological relevance as Ang III, Ang A. ANG (1–8) is a vital regulator of hemodynamic, but it has also been linked to tissue regeneration, remodeling, inflammation, and fibrosis.

Hypertension is found in 25–30% of the population of developed countries and is used as a conventional biomarker to increased cardiovascular risk [11,20]. Hypertension affects over 972 million people worldwide, and it has been estimated that patients with hypertension will account for 1.56 billion individuals by 2025 [21]. Monitoring blood pressure represents the most commonly used procedure to diagnose hypertension. Normal blood pressure is determined in a range of systolic blood pressure and diastolic pressure values of less than 120 mm Hg and 80 mm Hg. In the renin-angiotensin-aldosterone (RAAS) cascade signaling system, the generation of angiotensin I (Ang I) from the cleavage of angiotensinogen results in the production of Angiotensin II (Ang II) by the angiotensin-I converting enzyme (ACE), a dipeptidyl carboxypeptidase. Ang II is a key effector of the RAAS by exerting its biological functions via the two Ang II receptors: Ang II receptor type 1 (AT1R) and Ang II receptor type 2 (AT2R) (Figure 1). Ang II binds mainly through the activation of AT1R, responsible for arteriolar systemic vasoconstriction and inflammation in several tissues and cells [11]. Ang II can be hydrolyzed by ACE2 generating Ang1–7, which can bind the respective receptors, acting as ANG II receptors antagonist (Figure 1). ACE2 emerged as a potent negative regulator of the RAAS and inflammation.

In mouse models of ARDS, Ang II binding to Ang II receptor type 1a (AT1a) leads to impaired lung function and fibrosis, while treatment with an angiotensin receptor blocker (ARB) attenuates both inflammation and fibrosis. In a human study, elevated circulating Ang II concentrations in influenza A (H7N9) pneumonia were associated with higher mortality rates [22,23]. AT4 is an insulin-regulated membrane aminopeptidase (IRAP). In an alternative pathway, angiotensin-converting enzyme II (ACE2) converts Ang II to angiotensin (1–7), and angiotensin (1–10) is metabolized to angiotensin (1–9), which is a ligand for Angiotensin II receptor type 2 (AT2). ACE2 is a cell membrane-associated enzyme expressed on lung endothelial and epithelial cells, and is found in the heart and kidneys. Several studies showed that ACE2 deficiency leads to Ang II accumulation while reducing Ang (1–7) production. Ang (1–7) promotes wound healing, tissue regeneration, and reduces reactive oxygen species (ROS) by binding to its cognate receptor, Mas. Similar to Ang (1–7), Ang (1–9) also has regenerative and anti-inflammatory properties through its binding to AT2. Ang (1–10) is also a substrate of neprilysin (NEP) to form Ang (1–7). [22,23]

Systemic inflammation can compromise vasodilatation, leading to endothelial cell dysfunctions, which in turn triggers off thrombosis. Hypertension can be associated with insulin resistance, as well as obesity and diabetes [24]. Together with obesity and diabetes, hypertension accounts as a known risk factor for atrial fibrillation [20]. Hypertension is also the main cause of mortality by stroke. Nevertheless, the physio-pathological mechanisms governing hypertension have not been completely elucidated [20].

On the one hand, inflammation drives relevant structural changes in the left ventricle and left atrium [25,26,27]. Over-expression of Interleukin 6 (IL-6) and tumor necrosis factor-alpha (TNF-α) that regulate the production of high sensitivity C-reactive protein (hsCRP) are linked to hypertension and obesity [20]. High blood pressure is directly related to increased levels of hsCRP, which is produced in tissues undergoing chronic inflammation [28]. Inflammatory infiltrates, and damage related to the generation of reactive oxygen species (ROS) can up-regulate the expression of angiotensin II receptors, which in turn induces an increase in atrial cell death and infiltrations of leukocytes found in the arterial tissue [20,29].

## 3. Cardiovascular Active Peptides Endowed with ACE-Mediated Anti-Hypertension Activities

Several bioactive peptides of natural origin have shown antitumor, antidiabetic, anti-hypertensive, anti-hypocholesterolemic, and multifunctional properties [30]. Bioactive peptides could be defined as a “new generation” of bioactive regulators [31]. More than 3500 different bioactive peptides are reported in the Biopeptide database BIOPEP [32]. Sources of natural bioactive peptides include terrestrial, such as phytochemicals or animal-derived, as dietary products [33], or marine organisms, photosynthetic organisms such as terrestrial plants or algae [34], but also animal species, such as tropical amphibians [33], sea cucumbers [35], as well as unicellular organisms as cyanobacteria [35].

Among this large variety of defined peptides, some have been identified as competitive or non-competitive ACE inhibitors. The binding of the non-competitive inhibitors to the ACE alters the conformation of the ACE itself. Thus the enzyme, the substrate, and the inhibitor cannot form a complex [36]. The C-terminal end of specific inhibitory peptides binds to the active pockets of the ACE site. Many studies have shown that peptides with high ACE inhibitory activity contain Trp, Phe, Tyr, or Pro at C-terminal and aliphatic amino acids branched at N-terminal [36].

Active biopeptides can be generated by digestion, isolation, and purification processes, which influence the structure, composition, and features of the final product [37]. Production, isolation, purification, and characterization processes are expensive in terms of time, reagents, and facilities. Therefore, bioinformatics tools, such as BIOPEP, Antimicrobial Peptide Database (APD), and PepBank [38,39], have been implemented to quantitatively predict the structure–activity relationship for the investigated agents [40,41]. Table 1 shows marine-derived cardioactive peptides that can be classified into Renin-Inhibitory and ACE-Inhibitory [42] with their potency expressed as half-maximal inhibitory concentration (IC50) value [37].

Some discrepancies have been reported in studies investigating the ACE inhibitory effect of numerous polypeptides of marine origin in lowering blood pressure in vitro and in vivo [44]. This can be explained as a consequence of the different metabolic bio-transformations occurring in vitro versus in vivo. The ACE-i (ACE inhibitors) peptides must pass through the digestive tract and reach the intestinal epithelium so that, after oral administration, the ACE-i peptides sometimes have less effect on blood pressure than small synthetic molecules due to the hydrolysis they undergo due to pancreatic proteases, trypsin, chymotrypsin, elastase, and carboxypeptidases A and B [44,69,70,71].

Interestingly biopeptides with ACE-i activity, such as peptides derived from fish flake gelatin, resist the proteolytic environment characteristic of the gastric and the enteric environment related to their acid and basic pH, respectively [72]. The decrease in systolic blood pressure induced by fish-originated gelatin peptides was more significant than that caused by 2 mg/kg of the drug captopril from days 7–21. Although gelatin peptides dosage are higher than that of Captopril, the gelatin peptides from fish are an interesting natural alternative since obtained from food sources and could be consumed without toxic side effects [72]. Other studies comparing peptides isolated from the Nile tilapia *Oreochromis niloticus* (Linnaeus, 1758) with Captopril have shown anti-hypertensive effect without cytotoxicity in vitro by acting as a non-competitive inhibitor, unlike Captopril, which is a competitive inhibitor [73].

Although bioactive peptides are generally isolated from the original protein structure, some of them are also found free in natural sources [31]. Based on their biological and pharmaceutical properties, the production of bioactive peptides obtained by enzymatic hydrolysis [40], chemical synthesis [42], or microbial fermentation [74], has emerged as a relevant issue. Other manufacturing processes include separation and purification techniques, such as gel filtration, ultrafiltration [31], reverse phase ultra-reverse flow liquid chromatography (RP-UFLC) [31] and high-performance reverse-phase liquid chromatography (RP-HPLC), and characterization methods as ultra-performing liquid chromatography-tandem mass spectrometry (UPLC-MS/MS) [40].

Major drug classes are designed to target inflammatory processes and modulation pathways (RAAS blockers and statins) for the treatment of hypertension and related disorders [75]. Several drugs, such as beta-blockers, ACE inhibitors, angiotensin receptor blockers, calcium channel blockers, and aldosterone antagonists are prescribed to treat hypertension [76]. The papain hydrolysates, derived from the marine red alga *Palmaria palmata* (Linnaeus) Weber & Mohr, are defined as renin inhibitory peptides [47], inducing a strong reduction of blood pressure, following oral treatment of hypertensive rats [47].

Reducing the production of angiotensin II and RAAS activity (Figure 1) [20,77] is necessary to lower blood pressure. To reduce or avoid the onset of side effects associated with the use of anti-hypertensive drugs, the identification of novel compounds of natural origin still represents a very attractive field of investigation. For example, with the assumption of ACE inhibitors, the patient may experience severe or mild side effects, such as cough, headache, diarrhea, dizziness, fatigue, angioedema, and in rare cases, severe side effects, such as hyperkalemia (elevated levels of potassium) in rare renal and heart failure [78].

Many studies have shown that bioactive molecules from animals (terrestrial or marine) and photosynthetic organisms (plant or algae) could be used as anti-hypertension agents to treat cardiometabolic diseases [69,79,80,81,82]. Inhibition of ACE activity by dietary components occurs mainly through competitive inhibition [83]. Biopeptides with anti-ACE activities present in food can bind to the ACE and hinder its activity by altering blood pressure. Fang et al. [84] showed different mechanisms of inhibition of anti-hypertensive biopeptides using a computational model.

## 4. ACE2 in Cardiac Cell Homeostasis, Cardiac Remodeling, and Hypertension

ACE and ACE2 share 40% homology in their amino acidic sequence (Supplemental Figure S1), and they differ on their substrate specificities [85]. ACE2 acts as a monocarboxypeptidase, characterized by a single active site with higher affinity (~400-fold) for Ang II than Ang I [86], and is insensitive to ACE inhibitors (ACE-i) [22]. ACE2 expression is highly variable depending on tissue localization; elevated ACE2 expression levels are found in the kidney, lungs, intestine, heart, testis, adipose tissue, and endothelial cells [18,87]. ACE inhibitors and AT1R antagonists (ARBs) cause increasing ACE2 activity in the heart and kidneys in both normal and hypertensive rats [88,89,90]. The cleavage of the membrane-bound ectodomain of ACE2 by metalloproteinase ADAM17 releases soluble ACE2 into the plasma [91]. In a rat model of myocardial infarction, both ACE and ACE2 are increased in the border/infarct area compared with the viable area. [92]. Immunoreactivity of both ACE and ACE2 is augmented in failing human hearts [92]. The increase in ACE2 after MI suggests that it plays an important role in the negative modulation of the RAAS in the generation and degradation of angiotensin peptides after cardiac injury [92]. 

Decreased ACE2 expression could be a predisposing factor to hypertension. Several in vivo studies took advantage of the possibility to genetically manipulate ACE2 in the setting of pre-clinical models to study blood pressure and cardiovascular functionality. In ACE2 knockout mice, the increased blood pressure can accelerate cardiac dysfunction [93]. ACE2 is also crucial in regulating morpho/structural and functions of the heart. It has been shown that ACE2 knockout mice exhibit altered cardiomyocyte contractile capabilities, together with increased levels of Ang2 in plasma and cardiac tissues [94].

### ACE2 in Cardiac Remodelling and Hypertension

ACE2 is widely expressed in diverse cell populations within the heart, including cardiomyocytes, myofibroblasts, and coronary endothelial cells. The dynamic interactions between these cells, together with the inflammatory response, govern cardiac remodeling and adaptation following heart injury [25,95,96,97,98,99]. Cardiac remodeling, as a consequence of chronic hypertension or following an acute myocardial infarction, accounts as the major risk factor in heart failure development. RAAS acts as a relevant hub in orchestrating the progression of cardiac remodeling [23]. Inhibition of a hyperactive RAAS results in the protection against cardiac remodeling, limiting the risk of subsequent heart failure [62,100].

Overexpression of ACE2 has been reported to block cardiac remodeling and cardiac hypertrophy during the infusion of ANGII. Based on the reports that ACE2 acts as a crucial enzyme in the generation of ANG-(1–7), it could be hypothesized that the administration of ANG-(1–7) could prevent cardiac remodeling following chronic administration of ANG II. It has been reported that Sprague–Dawley rats receiving ANGII infusion exhibit increased blood pressure, myocyte hypertrophy, and midmyocardial interstitial fibrosis [62,100].

ACE2^−/−^ deficient mice display a phenotype similar to mice with cardioplegia, representing an adaptive response to coronary disease and bypass surgery [101]. These mice also show a higher expression of genes induced by hypoxia, a condition that causes vasoconstriction, endothelial dysfunction, and cardiac hypoperfusion [94].

Activated RAAS and Ang II have a crucial role as key mediators of hypertension. ACE2 is postulated to exert a potent activity as a blood pressure modulator, and therefore, ACE2 deficiency leads to hypertension. Recent studies confirm an association between ACE2 activity and blood pressure levels [102,103]: higher activity of circulating ACE2 has been found in serum or plasma of patients with hypertension as compared to healthy individuals [102,103]. In both male and female Type 1 diabetes patients with hypertension, serum ACE2 activity is correlated with systolic blood pressure [103]. These results clearly suggest that increased levels of ACE2 at tissue level act in attenuating response to hypertension [102,103]. Several pre-clinical models have been employed to investigate the anti-hypertensive activities of ACE2 [104,105]. Increased expression of RAAS anti-hypertensive components have been observed, following lentiviral overexpression of ACE2 and finally resulting in attenuation of high blood pressure [104,105]. Also, pre-treatment with rhACE2 resulted in decreased hypertension, following acute Ang II administration, together with decreased plasma Ang II and increased plasma Ang 1–7 levels [106]. The anti-hypertensive effects of ACE2/Ang 1–7 have generated increasing interest for their potential cardioprotective activities against hypertensive heart diseases, including HF, ischemic heart disease, hypertensive heart disease, and left ventricular hypertrophy.

## 5. Marine Cardiovascular Active Peptides Targeting the ACE System (ACE 1/ACE 2)

Currently, more than 125 ACE-inhibitory peptides have been identified in and isolated from marine organisms. The ACE inhibitory activity of marine-derived bioactive peptides appears to be higher than that of peptides from terrestrial sources [44]. Marine-derived ACE inhibitory peptides share a short aminoacidic sequence characterized by low molecular weights and hydrophobic residues [107] that confer increased anti ACE activities [37].

Fish-derived biopeptides, heterogeneous in terms of MW, length, and composition, have been reported to inhibit ACE activity and to successfully control blood pressure as revealed from in vitro and in vivo pre-clinical models, as well as in clinical studies [37].

Anti-ACE biopeptides isolated from fishes, similar to other bioactive inhibitors, exert anti-hypertension activities, without side effects associated with cytotoxicity, indicating them as a suitable alternative candidate of synthetic drugs for the treatment and prevention of hypertension, given the ability that they have shown to reduce blood pressure [37].

Concerning peptides from chum salmon *Oncorhynchus keta* (Walbaum, 1792), it was demonstrated that their activity is comparable to the pharmaceutical drug captopril [59].

Another source of ACE-inhibitor and antioxidant peptides can be generated from the hydrolysis of dietary proteins by trypsin and chymotrypsin [58,108]. For some sources of protein hydrolysates, such as those derived from the ascomycetous yeast *Kluyveromyces marxianus* (EC Hansen, Van der Walt), it seems essential that hydrolysates of trypsin and chymotrypsin undergo a further purification process to obtain ACE inhibitory activity compounds, with IC50 ranging between 15.20 mM and 22.88 mM, i.e., approximately 1.76 times to three times higher than the original peptide fraction [109].

Moreover, trypsinized hydrolysates of the Chinese venus *Cyclina sinensis* (Gmelin, 1791), after a series of separation and chromatographic purification steps, demonstrated enhanced ACE inhibitory activity. The new pentapeptide identified with the Trp-Pro-Met-Gly-Phe sequence (WPMGF; MW = 636.75 Da) showed potent ACE inhibitory activity and, interestingly, WPMGF kept this activity high (IC50 value of 0.789 mM) under pH values from 2 to 8 (simulating the conditions of the gastrointestinal environment) [110].

Another study reported a way to enhance the ACE-inhibitory activity of marine protein hydrolysates [111]. An example derived from the sea cucumber *Acaudina molpadioides* (Semper, 1867), when modified with 40% of exogenous proline (*w*/*w*, proline/free amino groups) using the plastein reaction, displayed higher ACE inhibitory activity than unmodified hydrolysates [111].

Another interesting ACE inhibitory effect is exerted by the two di- and tetrapeptides derived from the Asian kelp *Undaria pinnatifida* (Harvey) Suringar, which induce a decrease in blood pressure in hypertensive rats [59].

The seagrass *Halophila stipulacea* (Forsskål) (Ascherson 1867), native of the Indian Ocean and the Red Sea and invading the Mediterranean Sea, represents an example of a marine organism that is pharmacologically promising [74]. *Halophila stipulacea* exhibit different bioactivities, including antioxidant and anti-hypertensive properties. These inhibiting properties have been associated with bioactive peptides from Ribulose-1,5-bisphosphate carboxylase/oxygenase (RubisCO, E.C. 4.1.1.39) of plants [112,113]. RubisCO, a photosynthetic bifunctional multimeric enzyme, plays a role in photorespiration and carbon fixation in the Calvin cycle, with 30–50% of RubisCO being soluble and composed of eight large (56 kDa) and eight small (14 kDa) subunits [34,80]. The small RubisCO subunits contain high quantities of cationic and hydrophobic amino acids [34], while a bioactive dipeptide (Phe-Cys), able to block oxidative stress, was obtained by in-silico thermolysin hydrolysis from the large RubisCO subunit [114]. Some RubisCO-derived peptides exhibited opioid activity, performing G protein-coupled receptor ligand functions, representing a very important drug target [34]. 

## 6. ACE2 Inhibitors against SARS-CoV-2

The molecular link between the pathogenesis of SARS viruses and the role of the ACE-system in cardiac, pulmonary, and systemic damage of the infectious disease has been an issue of investigation since the advent of the first pandemic caused by SARS-Cov 1. The SARS-CoV surface spike protein binding to ACE2, its main receptor, reduces ACE2 expression [19,87], and the loss of ACE2 expression exits in severe acute respiratory syndrome and cytokine storm. The Spike-Fc protein can bind to bronchial epithelial cells, alveolar pneumocytes, endothelial cells, and several other targets. In particular, Spike-Fc is mainly localized where severe lesions occur, including inflammatory exudates [87].

**Figure 2:**
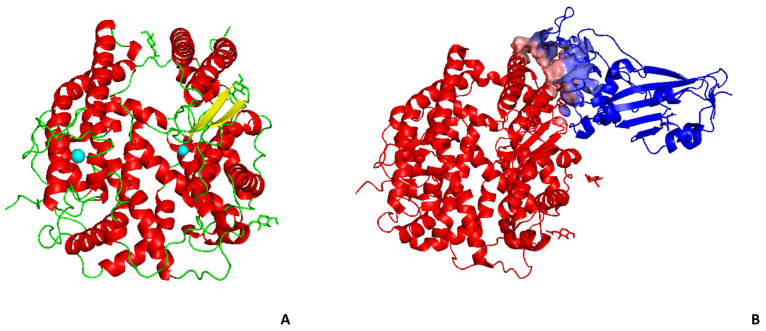
Molecular structure (ribbon representation) of human ACE2: (**A**) the ACE2 ectodomain, secondary structures are highlighted, in green loops, in red alpha-helices, in yellow beta-sheets, in cyan heteroatoms -Zn and C (PDB access code 1R42)l; (**B**) the ACE2-spike RBD complex, in pale cyan and pink the residues involved in the interface in the RBD and ACE2, respectively (PDB access code 6M0J).

The SARS-CoV Spike protein can directly influence the development of severe acute respiratory syndrome of the lung through ACE2 binding (Figure 2). Therefore, recombinant antagonist ACE2 protein, or derived peptides, may not only be a treatment to block the spread of SARS-CoV, but modulation of the RAAS could also be used to both protect people with SARS-CoV-2 and limit the spread of the current SARS-CoV-2 and other coronaviruses, by preventing the replication of the virus and the development of severe acute respiratory syndrome of the lung.

## 7. Molecular Docking with Human ACE2 and Interferences with SARS-CoV-2 Spike Protein

ACE2 inhibitors are not clinically relevant for hypertension, but with the SARS-CoV-2 pandemic, their use could pave the way to interfere with the binding of the Spike protein to ACE2. Molecular Docking (MD) is the elective methodology for the druggability screening of molecules towards target protein molecules [115]. This is based on the studies of molecular docking with a library of molecules; the studies suggest inhibition of the enzymatic activity of ACE2 with peptide analogs [54,65,116]. The N-(2-aminoethyl)-1 aziridine-ethanamide has good properties of inhibition of the activity of ACE2, by blocking membrane fusion with the SARS-CoV spike protein [103], but it also shows induction of hypertension and inflammatory activity [87]. To verify the druggability of peptides towards the activity of human ACE2, we tested the binding of three selected marine organism peptides of Table 2 in terms of molecular structure (Figure 2) and of complex stability (Table 3). We chose three peptides whose properties are reported in Table 2.

Figure 2 shows the molecular structure of the human ACE2 ectodomain (the part protruding from the cell membrane, thus accessible to ligand binding, PDB access code 1R42, chain A) and the human ACE2 bound to the spike protein RBD (Receptor Binding Domain) of SARS-CoV-2 (PDB access code 6M0J, in pale cyan and pale pink, are shown the residues involved in the interface in the spike RBD and ACE2, respectively).

The molecular structure of human ACE2 ectodomain shows a cleft splitting the two domains containing the heteroatoms (Zn and Cl in cyan in the ribbon structure, Figure 2). The cleft is liable to host binding peptides. The region involved in the SARS-CoV-2 spike protein binding is a small region (ten residues) highlighted in pink in Figure 1-panel B.

We performed molecular docking with HPEPDOCK (http://huanglab.phys.hust.edu.cn/hpepdock/), a web server for blind protein-peptide docking through a hierarchical algorithm [117].

**Figure 3:**
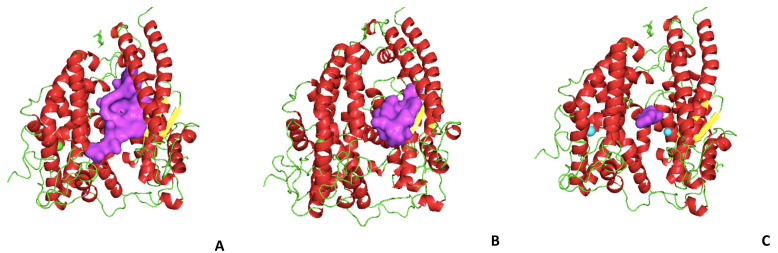
Molecular docking of selected peptides from Table 2 to human ACE2; in purple, the peptide configuration and position upon docking. (**A**) *Thunnus obesus*; (**B**) *Palmaria palmata*; (**C**) *Undaria pinnatifida*.

We present in Figure 3 the top conformations (scoring the highest structural stability) of ACE2 bound with the three peptides; in Table 3, the corresponding energy scores are reported.

**Figure 4:**
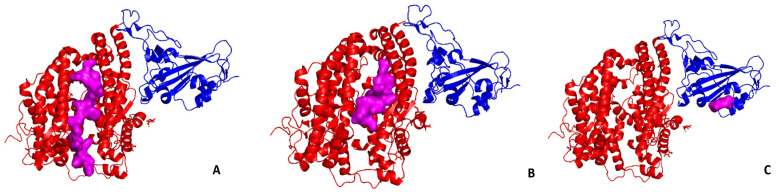
Molecular docking of selected peptides from Table 2 to the complex ACE2 – spike RBD; in purple, the peptide configuration and position upon docking. (**A**) *Thunnus obesus*; (**B**) *Palmaria palmata*; (**C**) *Undaria pinnatifida*.

Figure 4 reports the top solutions of the molecular docking of the peptides with the ACE2-spike RBD complex.

Table 2 shows the spike complex with respect to ACE2 (Figure 4 vs. Figure 3); in turn, the peptide from *Undaria pinnatifida* moves its binding region from the ACE2 cleft (Figure 4C) to the spike RBD.

Table 3 reports the energy scores for the complex of the peptides with the ACE2 and the ACE2-spike RBD.

The peptides from *Thunnus obesus* and *Undaria pinnatifida* form more stable complexes with ACE2 with respect to complexes with the spike-ACE2 form. On the contrary, the peptide binding from the *Palmaria palmata* to the spike-ACE2 is much more stable than that with ACE2.

It is also remarkable (see Figure 4C) that the peptide from *Undaria pinnatidica* moves its binding spot on the spike RBD, showing a strong affinity for it.

These preliminary results provide a molecular insight on the interaction of natural peptides with ACE2 and show that the effect of ACE2 drugs extends to the ACE2-spike complex. This aspect deserves further attention due to the possible synergistic action of ACE2 targeted drugs into the COVID19 outcome.

## 8. Conclusion and Future Perspectives

As cardiovascular diseases still remain the main cause of death worldwide, identifying novel bioactive compounds is of pivotal interest for an interventional clinical unmet need.

Marine biodiversity is a great source of biotechnological outputs, thanks to numerous peculiarities of organisms inhabiting seas and oceans. Biomedical applications are one of the branches that can take the most advantage of this only partially unexploited and largely under-investigated resource. New species, new molecules, or new productive pipelines could make natural compounds of marine origin available for human wellness and cures. Among the main illness-related causes of death worldwide, cardiovascular diseases are still the major killer. Hypertension is a widely present pathology. Although drugs for such disease do exist and fulfill their role, some of them may induce mild or severe side effects. Natural molecules are, therefore, a way to counteract these drawbacks.

A major question remains the reasons to explain a still limited development, particularly considering that most of these peptides are small, they are well within the range where chemical synthesis is possible, and they would represent a viable option for drug development. A possible explanation could be associated with the lack of strong intellectual property protection, given the natural origin of these peptides/products. A second related question is whether these are really candidates for therapeutic drug development or thwy should be used as a “nutraceutical” preventive supplement.

Here, we highlight how peptides from different marine organisms share valuable properties against cardiovascular morbidities, with only a few species over different unrelated phyla (from algae to fishes) investigated so far for such purpose. Given the great reservoir of marine species (from microorganisms to vertebrates), it is reasonable to hypothesize that many other peptides will display this bioactive property.

Therefore, more exploration is required in these peptides’ potential translational value, particularly for algae and/or microorganisms that can undergo eco-friendly production directly as nutraceuticals for human health.

The properties of these peptides to interfere with the RAAS system became even more important considering the new viral pandemic, the actual Covid-19, because the SARS-CoV-2 virus infection utilizes ACE2 as a receptor for viral entry. The pathogenicity and the severe prognosis of the Covid-19 infection is also enhanced by comorbidities with cardiometabolic diseases such as hypertension, diabetes, and heat failure.

The identification and design of new functional molecules, nutraceuticals and/or pharmaceuticals and bioactive compounds of marine source (alone or in combination with standard therapies), represents a big hope and a promising approach in both intervention and prevention of inflammatory cardiovascular diseases, hypertention and SARS viruses infection.

## Figures and Tables

**Table 1:** Cardiovascular active peptides from aquatic organisms/ACE Inhibitory peptides* modified from [15,43,44].

Phylum	Species	Amino Acid Sequence	IC_50_ (μM)	Ref.
Ochrophyta	*Undaria pinnatifida* (Harvey) Suringar	IY	2.7	[45]
		IW	1.5	
		VY	35.2	
		IY	6.1	
		AW	18.8	
		FY	42.3	
		VW	3.3	
		IW	1.5	
Rhodophyta	*Neopyropia yezoensis* (Ueda) Yang & Brodie (as *Porphyra yezoensis*)	LW	23.6	[46]
Rhodophyta	*Palmaria palmata* (Linnaeus) Weber & Mohr	LMPIIRLIIVLMA	3.344 × 10^3^	[47]
Arthropoda	*Acetes chinensis* Hansen, 1919	IFVPAFDP	3.4	[48]
		LHP	2.15	
Arthropoda	*Plesionika izumiae* Omori, 1971	ST	4.03	[49]
Mollusca	*Pinctada imbricata fucata* (Gould, 1850) (as *Pinctada fucata martensii*)	ALAPE	167.5	[50]
Cnidaria	*Rhopilema esculentum* Kishinouye, 1891	QPGPT	80.67	[14]
Annelida (Sipuncula)	*Phascolosoma arcuatum* (Gray, 1828) (as *Phascolosoma esculenta*)	AWLHPGAPKVF	135	[51]
Chordata (Pisces)	*Katsuwonus pelamis* (Linnaeus, 1758)	IKPLNY	43	[52]
		IVGRPRHQG	2.4	
		IWHHT	5.8	
		ALPHA	10	
		FQP	12	
		LKPNM	2.4	[52,53]
		IY	2.31	[52,53]
		DYGP	62	[52]
		LKP	0.32	[53]
		IWHHT	3.5	
		IKP	6.9	
		IVGRPR	300	[54]
Chordata (Pisces)	*Sarda lineolata* (Girard, 1858)	MF	44.7	[55]
		RY	51	
		MY	193	
		LY	38.5	
		YL	82	
		IY	10.5	
		VF	43.7	
		GRP	20	
		RFP	330	
		AKK	3.13	
		RVY	205.6	
		GWAP	3.86	
		KY	1.63	
		VY	10	[55,56]
Chordata (Pisces)	*Thunnus obesus* (Lowe, 1839)	GDLGKTTTVSNWSPPKYKDTP	11.28	[57]
		WPEAAELMMEVDP	21.6	[58]
Chordata (Pisces)	*Limanda aspera* (Pallas, 1814)	MIFPGAGGPEL	28.7	[59]
Chordata (Pisces)	*Gadus chalcogrammus* Pallas, 1814 (as *Theragra chalcogramma*)	GPM	17.13	[60]
		GPL	2.6	
		LGP	0.72	
		GLP	1.62	
		PLG	4.74	
		LPG	5.73	
		PGL	13.93	
		FGASTRGA	14.7	
Chordata (Pisces)	*Katsuwonus pelamis* (Linnaeus, 1758)	IKPLNY	43	[61]
		DYGLYP	62	
		LRP	1	
		LKPNM	2.4	
Chordata (Pisces)	*Sardinella aurita* Valenciennes, 1847	KW	1.63	[62]
Chordata (Pisces)	*Pseudocaranx* sp.	AR	570.78	[63]
		AV	956.28	
		APER	530.21	
		EY	1.98	
		FE	2.68	
		CF	1.45	
Chordata (Pisces)	*Saurida elongata* (Temminck & Schlegel, 1846)	RVCLP	175	[64]
		GMKCAF	45.70	
Chordata (Pisces)	*Thunnus albacares* (Bonnaterre, 1788) (as *Neothunnus macropterus*)	WGD	2	[57]
Chordata (Pisces)	*Oncorhynchus keta* (Walbaum, 1792)	WA	277.3	[65]
		VW	2.5	[65]
		WM	96.6	[65]
		MW	9.9	[42]
		IW	4.7	[65]
		LW	17.4	[65]
Chordata (Pisces)	*Oncorhynchus gorbuscha* (Walbaum, 1792)	IW	1.2	[15]
Chordata (Pisces)	*Okamejei kenojei* (Müller & Henle, 1841)	MVGSAPGVL	3.09	[66]
Chordata (Pisces)	*Alopias pelagicus* Nakamura, 1935	IKW	0.54	[67]
Chordata (Pisces)	*Scyliorhinus canicula* (Linnaeus, 1758)	VAMPF	0.44	[68]

**Table 2:** Properties of the peptides investigated in molecular docking. It is worth mentioning that small cationic-amphipathic peptides are likely to show antiviral properties, so peptides in Table 2 from *Thunnus obesus* and *Palmaria palmata* are also good candidates for counteracting COVID19 as antiviral peptides. Notice that the peptide from *Undaria pinnatifida* is also found in other organisms (see Table 1).

SOURCE	SEQUENCE	MW, g/mol	NET CHARGE pH 7.0	Average Hydrophilicity	% Hydrophilic Residues
*Thunnus obesus* (Lowe, 1839)	GDLGKTTTVSNWSPPKYKDTP	2292.53	1.0	0.2	38%
*Undaria pinnatifida* (Harvey) Suringar	VW	303.36	0	−2.5	0%
*Palmaria palmata* (Linnaeus) Weber & Mohr	LMPIIRLIIVLMA	1496.04	1.0	−1.1	8%

**Table 3:** Energy scores for the molecular docking top solutions. The ACE2 cleft appears to be a hot region, hosting the peptide in 5 out of 6 complexes.

Species	ACE2	ACE2-RBD
*Thunnus obesus*	−246.50	−223.60
*Palmaria palmata*	−245.44	−283.83
*Undaria pinnatifida*	−117.65	−123.42

## Supplementary Materials

The following are available online at https://www.mdpi.com/1422-0067/21/21/8364/s1, Figure S1: Sequences of: Angiotensin converting enzyme, Angiotensin converting enzyme 2, SARS CoV 2 spike glycoprotein

## Abbreviations

AGT	Angiotensinogen
ACE	Angiotensin I converting enzyme
ACE2	Angiotensin I converting enzyme 2
AngA	Angiotensin A
AP-A	Glutamyl aminopeptidase
AP-N	Alanyl aminopeptidase (membrane)
AT1R	Angiotensin II receptor type 1
AT2R	Angiotensin II receptor type 2
CMA1	Chymase 1
CPA3	Carboxypeptidase A3
CTSA	Cathepsin A
CTSG	Cathepsin G
IRAP	Leucyl and cystinyl aminopeptidase
MAS	MAS1 proto-oncogene, G protein-coupled receptor
MME	Membrane metallo-endopeptidase
MRGPRD	MAS related GPR family member D
NLN	Neurolysin
PRCP	Prolylcarboxypeptidase
PREP	Prolyl endopeptidase
PRR	ATPase H+ transporting accessory protein 2
RENRBD	ReninReceptor Binding Domain
THOP1	Thimet oligopeptidase
