# Supplementary material for: Cardiovascular Active Peptides of Marine Origin with ACE Inhibitory Activities: Potential Role as Anti-Hypertensive Drugs and in Prevention of SARS-CoV-2 Infection
Source: Int J Mol Sci. 2020 Nov 7;21(21):8364. doi: 10.3390/ijms21218364 (PMC7664667; doi:10.3390/ijms21218364)
Supplement: Supplementary file 1 [file ijms-21-08364-s001.pdf]

International Journal of
Molecular Sciences

MDPI

Supplementary Figure 1. Sequences of: Angiotensin converting enzyme, Angiotensin converting enzyme 2, SARS CoV 2 spike glycoprotein. Red area indicates the cleavage site of Furin, transmembrane protease Serine2 TMPRSS2. Green area indicates the binding site for the ACE2 peptide.

```
 Angiotensin-converting enzyme

        1 mgqgwatagl psllflllcy ghpllvpsqe asqqvtvthg tssqattssq ttthqatahq
       61 tsaqspnlvt deaeaskfve eydrtsqvvw neyaeanwny ntnittetsk illqknmqia
      121 nhtlkygtqa rkfdvnqlqn ttikriikkv qdleraalpa qeleeynkil ldmettysva
      181 tvchpngscl qlepdltnvm atsrkyedll wawegwrdka grailqfypk yvelinqaar
      241 lngyvdagds wrsmyetpsl eqdlerlfqe lqplylnlha yvrralhrhy gaqhinlegp
      301 ipahllgnmw aqtwsniydl vvpfpsapsm dtteamlkqg wtprrmfkea ddfftslgll
      361 pvppefwnks mlekptdgre vvchasawdf yngkdfrikq cttvnledlv vahhemghiq
      421 yfmqykdlpv alreganpgf heaigdvlal svstpkhlhs lnllsseggs dehdinflmk
      481 maldkiafip fsylvdqwrw rvfdgsitke nynqewwslr lkyqglcppv prtqgdfdpg
      541 akfhipssvp yiryfvsfii qfqfhealcq aaghtgplhk cdiyqskeag qrlatamklg
      601 fsrpwpeamq litgqpnmsa samlsyfkpl ldwlrtenel hgeklgwpqy nwtpnsarse
      661 gplpdsgrvs flgldldaqq arvgqwlllf lgiallvatl glsqrlfsir hrslhrhshg
      721 pqfgsevelr hs

Angiotensin-converting enzyme 2

        1 msssswllls lvavtaaqst ieeqaktfld kfnheaedlf yqsslaswny ntniteenvq
       61 nmnnagdkws aflkeqstla qmyplqeiqn ltvklqlqal qqngssvlse dkskrlntil
      121 ntmstiystg kvcnpdnpqe clllepglne imansldyne rlwaweswrs evgkqlrply
      181 eeyvvlknem aranhyedyg dywrgdyevn gvdgydysrg qliedvehtf eeikplyehl
      241 hayvraklmn aypsyispig clpahllgdm wgrfwtnlys ltvpfgqkpn idvtdamvdq
      301 awdaqrifke aekffvsvgl pnmtqgfwen smltdpgnvq kavchptawd lgkgdfrilm
      361 ctkvtmddfl tahhemghiq ydmayaaqpf llrnganegf heavgeimsl saatpkhlks
      421 igllspdfqe dneteinfll kqaltivgtl pftymlekwr wmvfkgeipk dqwmkkwwem
      481 kreivgvvep vphdetycdp aslfhvsndy sfiryytrtl yqfqfqealc qaakhegplh
      541 kcdisnstea gqklfnmlrl gksepwtlal envvgaknmn vrpllnyfep lftwlkdqnk
      601 nsfvgwstdw spyadqsikv rislksalgd kayewndnem ylfrssvaya mrqyflkvkn
      661 qmilfgeedv rvanlkpris fnffvtapkn vsdiiprtev ekairmsrsr indafrlndn
      721 sleflgiqpt lgppnqppvs iwlivfgvvm gvivvgivil iftgirdrkk knkarsgenp
      781 yasidiskge nnpgfqntdd vqtsf
```

SARS-CoV-2 spike glycoprotein

```
   1 mfvflvllpl vssqcvnltt rtqlppaytn sftrgvyypd kvfrssvlhs tqdlflpffs
  61 nvtwfhaihv sgtngtkrfd npvlpfndgv yfasteksni irgwifgttl dsktqslliv
 121 nnatnvvikv cefqfcndpf lgvyyhknnk swm esefrvy ssannctfey vsqpflmdle
 181 gkqgnfknlr efvfknidgy fkiyskhtpi nlvrdlpqgf saleplvdlp iginitrfqt
 241 llalhrsylt pgdsssgwta gaaayyvgyl qprtfllkyn engtitdavd caldplsetk
 301 ctlksftvek giyqtsnfrv qptesivrfp nitnlcpfge vfnatrfasv yawnrkrisn
 361 cvadysvlyn sasfstfkcy gvsptklndl cftnvyadsf virgdevrqi apgqtgkiad
 421 ynyklpddft gcviawnsnn ldskvggnyn ylyrlfrksn lkpferdist eiyqagstpc
 481 ngvegfncyf plqsygfqpt ngvgyqpyrv vvlsfellha patvcgpkks tnlvknkcvn
 541 fnfngltgtg vltesnkkfl pfqqfgrdia dt tdavrdpq tleilditpc sfggvsvitp
 601 gtntsnqvav lyqdvnctev pvaihadqlt ptwrvystgs nvfqtragcl igaehvnnsy
 661 ecdipigagi casyqtqtns pgsassvasq siiaytm slq aensvaysnn siaiptnfti
 721 svtteilpvs mtktsvdctm yicgdstecs nlllqygsfc tqlnraltgi aveqdknt qe
 781 vfaqvkqiyk tppikdfggf nfsqilpdps kpskrsfied llfnkvtlad agfikqygdc
 841 lgdiaardli caqkfngltv lpplltdem i aqytsallag titsgwtfga gaalqipfam
 901 qmayrfngig vtqnvlyenq klianqfnsa igkiqdslss tasalgklqd vvnqnaqaln
 961 tlvkqlssnf gais svlndi lsrldppeae vqidrlitgr lqslqtyvtq qliraaeira
1021 sanlaatkms ecvlgqskrv dfcgkgyhlm sfpqsaphgv vflhvtyvpa qeknfttapa
1081 ichdgkahfp regvfvsngt hwfvtqrnfy epqiittdnt fvsgncdvvi givnntvydp
1141 lqpeldsfke eldkyfknht spdvdlgdis ginasvvniq  keidrlneva knlneslidl
1201 qelgkyeqgs gyipeaprdg qayvrkdgew vllstflgrs levlfqgpgh hhhhhhsaw
1261 shpqfekggg sggggsggsa wshpqfek
```

QVAV 610
LYQDV 615
LYQGV 615
NCTEV 620

The site in red is the cleaving point of Furin, transmembrane protease serine 2 TMPRSS2…

The site in green is the binding point of the ACE2 peptide, and other am ino acids.
